# Influence of intersignaling crosstalk on the intracellular localization of YAP/TAZ in lung cells

**DOI:** 10.1186/s12964-024-01662-2

**Published:** 2024-05-27

**Authors:** I. A. Govorova, S. Y. Nikitochkina, E. A. Vorotelyak

**Affiliations:** Koltsov Institute of Developmental Biology, Russian Academy of Sciences, Vavilov str, 26, Moscow, 119334 Russia

**Keywords:** Hippo, YAP, TAZ, WNT, SHH, TGFβ, Notch, mTOR, Lung epithelium, Lung mesenchyme

## Abstract

A cell is a dynamic system in which various processes occur simultaneously. In particular, intra- and intercellular signaling pathway crosstalk has a significant impact on a cell’s life cycle, differentiation, proliferation, growth, regeneration, and, consequently, on the normal functioning of an entire organ. Hippo signaling and YAP/TAZ nucleocytoplasmic shuttling play a pivotal role in normal development, homeostasis, and tissue regeneration, particularly in lung cells. Intersignaling communication has a significant impact on the core components of the Hippo pathway and on YAP/TAZ localization. This review describes the crosstalk between Hippo signaling and key lung signaling pathways (WNT, SHH, TGFβ, Notch, Rho, and mTOR) using lung cells as an example and highlights the remaining unanswered questions.

## Introduction

The signaling pathways that regulate different processes of homeostasis and regeneration in organs and tissues mutually mediate each other. In mammalian airway cells, the most discussed pathways are Hippo, Wingless-related Integration Site (WNT), Sonic Hedgehog (SHH), Transforming Growth Factor β (TGFβ), Bone Morphogenetic Protein (BMP), Notch, and Mammalian Target of Rapamycin (mTOR) signaling pathways [1–6 and others]. Each of these signaling pathways is pivotally important for the normal development and functioning of the lungs. In airway system research, the Hippo signaling pathway is of particular interest due to its involvement in regulating cell fate, proliferation, differentiation, and interactions between epithelial, mesenchymal, and endothelial cells, as well as tissue regeneration at every stage of lung development. Furthermore, dysregulation of the Hippo cascade can result in airway system pathologies. The Hippo pathway is regulated through interactions with other signaling pathways as well as through cytoskeletal mechanical tension. Additionally, mechanotransduction is an important feature of the Hippo pathway transcriptional co-factors yes-associated protein (YAP) and PDZ-binding motif (TAZ). Furthermore, the nucleocytoplasmic shuttling of YAP/TAZ is crucial for the branching processes and normal development of proximal and distal lung compartments [5, 7]. Therefore, the regulation of YAP/TAZ intracellular localization is influenced by the intersignaling crosstalk and cytoskeletal tension. However, a comprehensive explanation of the influence of interpathway crosstalk on YAP/TAZ nucleocytoplasmic shuttling under biologically relevant conditions that do not cause artificial cytoskeletal deformation has yet to be provided. This review focused on describing the mechanisms of mutual regulation between the Hippo cascade and the WNT, Notch, SHH, TGFβ, and mTOR signaling pathways in the lungs while emphasizing existing research gaps. In addition, special attention has been given to the influence of intersignaling crosstalk on intracellular YAP/TAZ localization in different types of mammalian lung cells. This review consists of two main subdivisions, describing (1) the Hippo signaling pathway in mammalian lung cells and the role of YAP/TAZ in airway development and pathologies and (2) the impact of mutual regulation between the Hippo cascade and the WNT, Notch, SHH, TGFβ, and mTOR signaling pathways on YAP/TAZ localization in mammalian lung cells. Comprehensive insights into YAP/TAZ signaling regulation in lung cells are necessary for a better understanding of normal functioning and the mechanisms of development and pathogenesis and may be used to define novel approaches for the treatment of airway diseases.

## HIPPO-YAP signaling in lung cells

### Background

The lung cellular architecture has been well described and continues to be described in detail at single-cell resolution [8]. Distal airways include basal cells characterized by the expression of *TP63, KRT5*, and *NGFR*; club cells characterized by the expression of *SCGB1A1* and *SCGB3A2*; goblet cells characterized by the expression of *MUC5AC, FOXA3*, and *SPDEF*; ciliated cells characterized by the expression of *FOXJ1* and *b-tubulin IV*; NE cells characterized by the expression of *ASCL1* and *CALCA*; ionocytes characterized by the expression of *FOXI1* and *CFTR;* and tuft cells characterized by the expression of *TRPM5* and *GNG13*. Proximal alveoli include alveolar cells type 1 – AT1 (*PDPN, AGER, HOPX, AQP5*) and type 2 – AT2 (*SFTPC, DC-LAMP*) [9]. In addition to different epithelial cells, there is a large number of mesenchymal cell types that are equally important in normal homeostasis and lung functioning [10]. However, the mechanisms by which the mesenchymal microenvironment influences different epithelial cells, lung development and regeneration and which signaling pathways are important in these interactions remain unclear.

The Hippo signaling pathway is an important participant in lung cellular homeostasis and is described in detail in both healthy and pathological states [11–13]. The transcriptional coactivators of the Hippo pathway are YAP and its homolog TAZ; they regulate target gene expression and, as a consequence, proliferation, differentiation, and cell viabilty. An important feature of YAP/TAZ proteins is their dynamic changes in intracellular localization or nucleocytoplasmic shuttling. The intracellular localization of YAP/TAZ influences the maintenance of cell stemness, differentiation, and proliferation and depends on the Hippo pathway interactions with other signaling pathways [5]. However, the numerous factors regulating the YAP/TAZ nucleocytoplasmic shuttling in cells, such as mechanotransduction, intersignaling and cell-cell interactions, require additional research on biologically relevant models.

The canonical way to control YAP/TAZ nucleocytoplasmic shuttling is through the Hippo signaling pathway. Briefly, the active Hippo cascade starts with MST1/2 kinase phosphorylation by TAO kinase, followed by MST1/2 binding with the SAV1 and Mob1A/B proteins via the C-terminal SARAH domain. After that, the MST1/2-SAV1 complex phosphorylates and activates LATS1/2 kinase, which inhibits YAP/TAZ via “inactivating” phosphorylation by the Ser61, Ser109, Ser127, Ser164, and Ser381 amino acid residues. When YAP is phosphorylated at Ser127, it induces YAP sequestration into the cytoplasm through the 14–3–3 sigma protein. The nonactive Hippo pathway causes YAP/TAZ binding to Tyr357 and Tyr316 and translocation of the complex to the cell nucleus. Nuclear YAP/TAZ forms a complex with DNA-binding transcription factors (TEADs 1–4) and regulates the expression of target genes, such as connective tissue growth factor (*CTGF*) and cysteine-rich angiogenic inducer 61 (*CYR61*).

### The role of YAP/TAZ mechanotransduction in lung

The subcellular localization of YAP/TAZ depends on the cytoskeletal tension, indicating that mechanotransduction is an important function of the YAP/TAZ proteins. The actin cytoskeleton and cell mechanical tension are mediated by actin filaments through the Rho family of GTPases and the GPCR signaling pathway. When GPCR signaling is active, Gα proteins regulate YAP/TAZ localization via Rho, Cdc42, Rac and Ras GTPases, participate in LATS1/2 inhibition, and stabilize F-actin filaments. Therefore, F-actin accumulates in cells, inhibits the Hippo pathway, reduces YAP phosphorylation and promotes nuclear YAP accumulation. Cdc42/F-actin/MAPK/YAP is critical for the polymerization of actin filaments and regeneration processes in AT2 cells of mouse lungs [14]. Deletion of Cdc42 and mechanical tension activate the TGFβ signaling cascade in AT2 cells and promote lung fibrosis progression [15]. An in vitro study on the human alveolar epithelial cell line BEAS-2B showed that the YAP/F-actin/MAPK cascade plays an important role in lung cell tensile overload and injury [16].

**Fig. 1:**
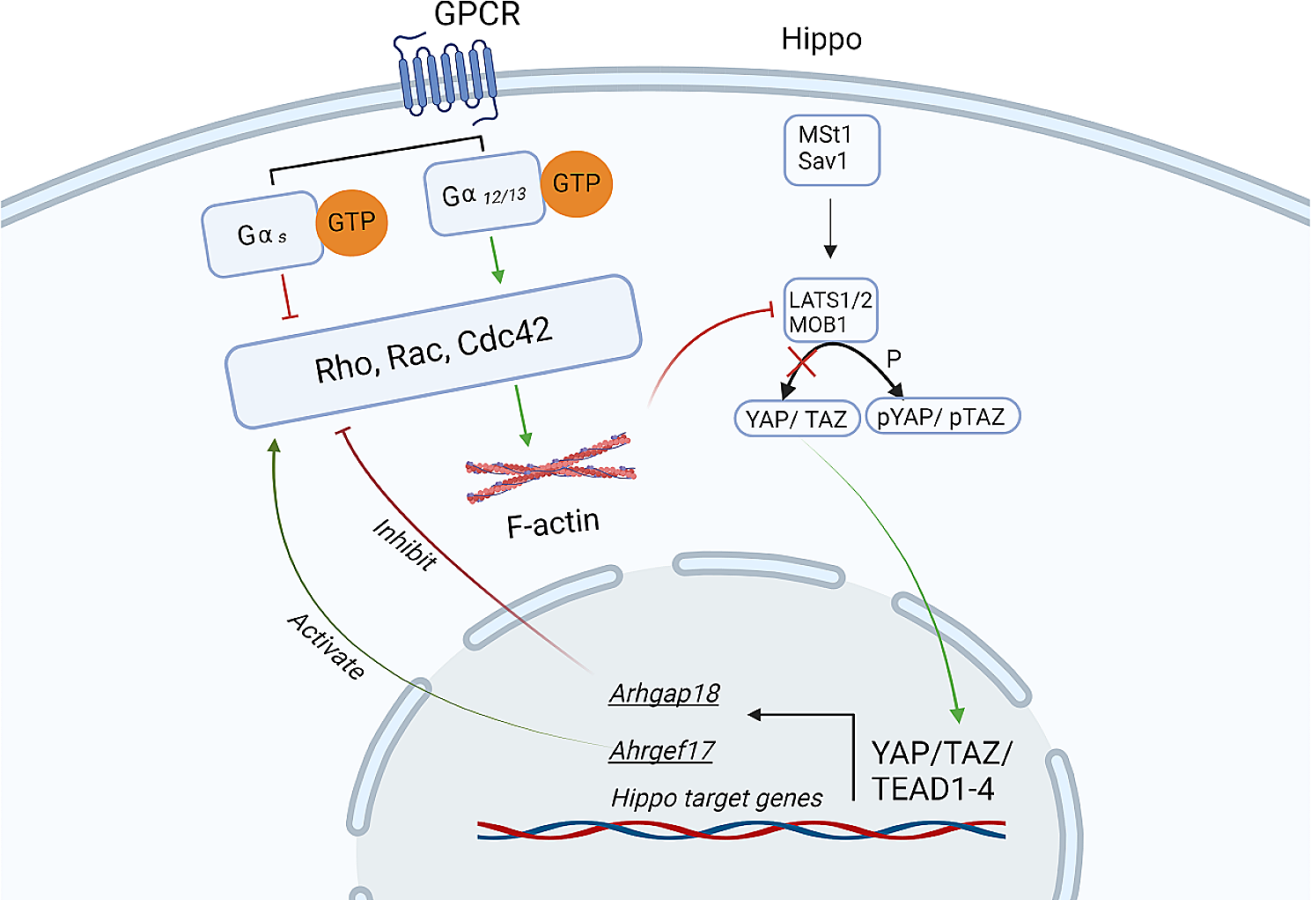
Schematically presented “feedback loop” between nuclear YAP/TAZ and Rho GTPases. Context-dependent activation/inactivation of cell cytoskeletal tension via the Hippo pathway. The figures were created with BioRender.com

Moreover, as mentioned in [5], there is a poorly described feedback loop of the influence of YAP/TAZ intracellular localization on the mechanisms of cytoskeletal organization and cell polarity that lead to the stretching and flattening of cells. In fact, in flat and elongated AT1 cells, YAP is predominantly localized in the nucleus, but in cuboidal AT2 cells, YAP is mostly detected in the cytoplasm [17, 18]. Furthermore, the deletion of YAP in alveolar cells decreased the number of AT1 cells and increased the number of AT2-like cells in the lungs [18–21]. A study by Lin et., 2017 [22] provide strong evidence that nuclear localization of YAP promotes mechanical tension via myosin light and heavy chains in lung distal bud tips, which ensures normal bronchial morphogenesis, through the YAP–RhoGEF–RhoA–ROCK cascade activation via the *Arhgef17* gene (Fig. 1). Also, YAP controls tissue tension and maintains alignment required for three-dimensional body shape in vertebrates via the ARHGAP18 gene, which suppresses actin polymerization [23]. Furthermore, ARHAGP18 overexpression leads to inhibition of RhoA and interrupts stress fiber formation [24]. Thus, YAP can regulate mechanical tension and change cell shape through activation or inhibition of the Rho pathway. The exact circumstances under which YAP induces the activation or inhibition of Rho signaling remain unclear.

### YAP/TAZ in lung development

Hippo/YAP signaling is pivotally important for normal lung cell development, function and regeneration. Lung development in mice can be divided into several stages: embryonic (E9–E11.5), pseudoglandular (E11.5–E16.5), canalicular (E16.5–E17.5), saccular (E17.5–P5) and alveolar (P5–P30) stages. During the embryonic and pseudoglandular stages, the formation of tracheal and lung buds, major bronchi branching, vasculogenesis and angiogenesis, and epithelial proliferation are stimulated by fibroblasts. During the canalicular stage, active vascularization and angiogenesis continue, terminal bronchioles are divided into respiratory bronchioles and alveolar ducts, and the epithelium is differentiated into squamous distal and cuboidal proximal cells. During the saccular stage, a lymphatic network develops in the lung; mesenchyme promotes differentiation of the alveolar epithelium; primary septae form; epithelial cells express surfactant; and fetal lungs are able to support gas exchange. The final stage of lung development is the alveolar stage, where alveolarization, growth and septation of the alveoli, and maturation of the vascular system in the lungs occur [25–27].

The findings of [28] demonstrated that during different stages of lung embryogenesis, the homologs YAP and TAZ, despite their similarities, participate in separate processes. In the pseudoglandular stage, YAP is mostly expressed in the bronchi. Deletion of YAP in murine lung cells at this stage leads to small cystic lungs and obstructive airway development. At the saccular and early alveolar stages, TAZ expression prevails in lung epithelial cells but not in mesenchymal cells. In addition, conditional TAZ knockout in lung cells at these stages results in emphysema-like lungs. Furthermore, TAZ deficiency in epithelial AT2 cells leads to disruption of alveolar epithelial cell differentiation [29]. These findings indicate that YAP is essential for bronchial morphogenesis and that TAZ is essential for early alveolarization of the lung. Moreover, YAP/TAZ and its intracellular localization plays a compartment-specific role in normal branching morphogenesis and lung proximal-distal development [7, 18]. In [7] shown a critically important role of YAP nucleocytopasmic shuttling for the expression of Sox2 in airway epithelial progenitors in early embryogenesis. Active Yap results in high Sox2 expression and differentiation of airway epithelial progenitors. However, subsequent research of [18] demonstrated that nuclear YAP is required in the Sox9 + progenitor compartments (but not in the Sox2 + airway compartment) to form distal branches and initiate airway morphogenesis. Furthermore, nucleocytoplasmic shuttling itself is not required for alveolocytes differentiation, but is crucial for airway epithelium differentiation in embryogenesis. In a lipopolysaccharide (LPS)-induced inflammation model at the pseudoglandular stage of lung development, YAP/TAZ phosphorylation is activated in fetal lung fibroblasts, leading to abnormal lung branching [30]. Additionally, the intracellular localization of YAP regulates basal cell differentiation during normal lung development [7, 18]. Nuclear YAP/TAZ in the airway epithelium regulates goblet cell differentiation and prevents mucin overexpression, which is a typical feature of bronchitis, COPD and asthma [31]. In embryonic (E18.5) and postnatal murine lungs, YAP regulates the proliferation and differentiation of airway epithelial cells via the Ajuba LIM protein [32]. In postnatal and adult murine lungs, active or nuclear YAP increases AT1 cell numbers and regulates alveolar epithelial cell (AT1/AT2) differentiation [20, 21]. Moreover, YAP/TAZ deletion decreased AT1 but not AT2 cell numbers [17]. In human in vitro cultivated iPSCs, nuclear YAP initiates a transcriptomic shift from PSC-derived AT2s (iAT1s) to iAT1 cells [19].

Therefore, the balance of active and phosphorylated YAP/TAZ is crucial for normal lung development, and disruption of the YAP/TAZ nucleocytoplasmic shuttling dynamic causes diverse lung pathologies during both embryogenesis and the postnatal/adult stage. However, the exact mechanisms by which YAP/TAZ influences differentiation processes and the maintenance of homeostasis in distinct lung cell populations at different stages of development have not been fully described. It is important to note that the Hippo pathway is as significant in the lung epithelium as it is in surrounding mesenchymal cells, and the precise mechanisms of YAP/TAZ regulation in mesenchymal cells have been investigated too. Such, active YAP/TAZ in myofibroblasts was associated with fibrosis in different organs [33], specifically nuclear YAP/TAZ are key regulators of pathological fibroblast activation in lung fibrosis [34]. Also, work on lung fibroblasts has shown that YAP/TAZ inhibition through dopamine receptor D1 [35] and GPCR-mediated cAMP signaling [36] reversed abnormal fibroblast activation and tissue fibrosis. Furthermore, both YAP and TAZ activity is required to control the fibroblast contractile function [37]. However, precise mechanisms of YAP/TAZ regulation in mesenchymal cells in developing lungs have not been fully investigated and remain virtually unexplored.

### Hippo pathway dysregulation in lung cells

Hippo pathway dysregulation can cause the development of different lung pathologies, such as idiopathic pulmonary fibrosis, asthma, chronic obstructive lung disease, bronchitis, pneumonia, pulmonary arterial hypertension, and lung cancer.

In particular, in the developing lung epithelium, MST1/2 knockout leads to the uncontrolled differentiation of AT2 cells and decreased surfactant protein production [38]. Inactivation of LATS1/2 kinase in developing murine lungs (E10.5–18.5) results in disruption of branching morphogenesis and an increased number of stretched AT1 and basal cells [17]. Null mutations of the Mob1A/B protein in embryonic lungs contribute to increased proliferation of alveolar cells and decreased surfactant protein expression [39]. MST1/2 kinase deletion in primary human bronchial cells in vitro inhibits the differentiation of secretory, goblet, and ciliated cells [32]. YAP/TAZ inactivation after acute injury leads to continual alveolar inflammation and fibrosis [40]. YAP/TAZ deletion in the lungs provokes serious abnormalities in lung structure, lung inflammation, mucin gene overexpression, goblet cell metaplasia, and deprivation of AT1 cell genes, all of which cause high mortality [31]. However, the effects of the dysregulation of other Hippo pathway components on lung development are poorly described.

Furthermore, the influence of Hippo signaling on cell processes has been demonstrated in different models of lung pathologies. For example, in response to fetal tracheal occlusion, Hippo/YAP signaling in the lung epithelium is activated and is responsible for increasing the number of basal cells [41]. In response to *S. pneumoniae* strain T4 (SpT4) lung infection, YAP/TAZ is activated in AT2 cells, and YAP/TAZ deletion in AT2 cells impairs alveolar epithelial regeneration and promotes fibrosis [42]. In addition, nuclear YAP/TAZ bound to TEAD transcription factors can bind to the TP63 factor and form a YAP/TAZ-TEAD-TP63 complex, which downregulates genes associated with early immune evasion and contributes to lung carcinogenesis, as has been shown in human bronchial cells [43].

In summary, Hippo/YAP signaling is pivotally important for the normal development, function and repair of the lung (Table 1). Under physiological conditions, YAP/TAZ can be partly present in the cytoplasm and nucleus, and its localization depends not only on Hippo kinase activity but also on Hippo–YAP/TAZ interactions with components of other pathways and extracellular and intracellular processes such as cell–cell and cell–matrix interactions, cell polarity, cellular mechanotransduction, inflammation and metabolism. This review focused on Hippo signaling interactions with key components of different pathways in the lungs. Intersignaling communications are important to consider in the context of a detailed investigation of YAP/TAZ shuttling in live cells.

**Table 1:** YAP/TAZ regulation in lung cells

	Normal YAP/TAZ regulation	YAP/TAZ overexpression	YAP/TAZ deletion
*Alveoli*	Controls normal AT2-AT1 differentiation after lungs injury [20, 21, 29]; alveoli growth and development	Increases numbers of AT1 cells [17, 18]; increases AT1 cells differentiation [20, 21]; promotes loss of the AT2 cells program [19]	Promotes spontaneous reprogramming of AT1 cells into the AT2 cell lineage [18, 20]; results in emphysema-like lung phenotype with increased number of inflammatory cells [28, 44]
*Bronchi*	Regulates branching morphogenesis; basal cell differentiation; proliferation of airway epithelial cells	Induces club cell proliferation; airway epithelial hyperplasia in nonciliated bronchiolar epithelial cells [32]; cancerogenesis [43]	Disrupts bronchial development [28]; mucin gene hypersecretion, goblet cell metaplasia [31]

### Intersignaling crosstalk in lung cells

#### WNT-Hippo

Hippo and WNT signaling pathway interactions play an important role in the investigation of cellular developmental mechanisms and airway functioning [45]. The WNT signaling pathway is well studied and is divided into canonical and noncanonical (alternative) WNT pathways. In early lung embryogenesis (E9.5–E12.5), the canonical WNT/β-catenin pathway causes mesenchymal-to-endodermal crosstalk that is critical for bud tip development from tracheal-esophageal septation [46]. During the pseudoglandular and canalicular stages (E12.5–E16.5) of murine lung development, the Wnt2a protein and endodermal-derived Wnt7b, which are activated via FGF9 signaling, promote and support mesenchymal cell proliferation and pulmonary vasculature growth [46, 47]. At the early and late alveolarization stages (P4-P21), epithelial WNT-dependent Axin2 + AT2 cells were detected; these cells regulate alveologenesis and control AT2-AT1 differentiation via the WNT/β-catenin cascade [46, 48]. Furthermore, WNT-dependent Wnt2+/PDGRDα + and Axin2+/PDGRDα + subpopulations of mesenchymal niche cells were found in the alveolar region of adult murine lungs and are important for regeneration after acute lung injury [49]. Additionally, distinct subpopulations of LGR5 + and LGR6 + PDGRDα + mesenchymal cells, which may play a role in alveolar and airway diseases, respectively, have been described [50]. In particular, the ligand Wnt3a modulates LGR5 + mesenchymal cells and participates in alveolar lineage differentiation.

Spatiotemporal interactions between the Hippo and WNT pathways in cells can play a crucial role in tissue homeostasis and self-renewal. In vitro experiments on cell cultures of HEK-293T, H1299, and A549 cells showed that the active Hippo pathway can suppress WNT/β-catenin signaling directly through MST1/2 kinase [51]. MST1/2 can bind to casein kinase 1ε (CK1ε), a protein that regulates the β-catenin degradation complex. Moreover, MST1/2 kinase, via phosphorylation of TAZ, can interact with DVL2 and disturb the DVL2-CK1ε complex, thereby inhibiting the Wnt3a-induced phosphorylation of DVL2, which in turn leads to the suppression of WNT/β-catenin signaling [52]. In murine in vivo and human in vitro models of intestinal tumorigenesis, Hippo-WNT/β-catenin crosstalk has been shown to occur via adenomatous polyposis coli (APC), a downstream effector of the canonical WNT pathway, acting as a scaffold protein for the SAV1 protein and LATS1/2 kinase [53, 54].

WNT/β-catenin can also directly influence the intracellular localization of YAP/TAZ. Active WNT/β-catenin signaling inhibits cytoplasmic YAP/TAZ, stabilizes β-catenin and promotes nuclear YAP/TAZ localization, as has been demonstrated in vitro in HEK293 cells, ST-2 mesenchymal stem cells, and P19 embryonic carcinoma cells [55]. Inside the nucleus, YAP/TAZ can bind to β-catenin, forming a YAP/β-catenin/TCF transcriptional complex [56]. In the case of the inactive WNT/β-catenin pathway, the E3 ubiquitin ligase β-TrCP can recognize both cytoplasmic YAP/TAZ and β-catenin and cause β-catenin and YAP/TAZ degradation. Two major mechanisms of β-catenin degradation have been described. First, the cytoplasmic destruction complex captures β-catenin by phosphorylating CK1 and GSK3, activating the process of β-catenin degradation. The second is caused by the Hippo-WNT pathway interaction: cytoplasmic YAP/TAZ binds to the destruction complex via Axin, followed by Axin/YAP/TAZ complex recruitment of the β-TrCP ligase to decrease phospho-β-catenin levels and inhibit WNT/β-catenin. In fact, cytoplasmic YAP/TAZ can bind with Axin via the same domain as the LRP6 receptor; however, in the case of active WNT/β-catenin signaling, the Axin/LRP6 complex interaction dominates, and YAP/TAZ is displaced from the β-catenin destruction complex. In addition, the “WW” domain of TAZ can specifically bind with β-catenin, and the phospho-β-catenin/TAZ/β-TrCP complex causes simultaneous degradation of β-catenin and TAZ [57]. Active Hippo signaling inactivates YAP/TAZ in the cytoplasm through phosphorylation at Ser127, which suppresses β-catenin/TCF/LEF transcriptional activity and downstream gene expression, such as that of MMPs and c-Myc, as shown in vitro in Caco-2, HT-29, and HEK293T cells [58, 59]. At the same time, WNT/β-catenin pathway inhibitors, including DKK1, BMP4, and IGFBP4, are YAP/TAZ-TEAD target genes, so nuclear YAP/TAZ localization can suppress canonical WNT signaling via the prevention of β-catenin nuclear localization [60]. Therefore, YAP/TAZ are a significant part of the WNT/β-catenin destruction complex, and their intracellular localization changes depending on the activation or inactivation of WNT signaling. Active WNT/β-catenin leads to YAP/TAZ localization in the cell nucleus, while inactive WNT/β-catenin leads to cytoplasmic YAP/TAZ localization (Fig. 1).

The noncanonical (alternative) WNT pathway includes planar cell polarity (PCP) and the WNT/Ca2 + pathways that regulate cell polarity and migration, as well as mechanisms of regeneration and cancerogenesis. Noncanonical WNT signaling can partially dysregulate the canonical WNT/β-catenin pathway, which mediates the normal functioning of the lung alveolar epithelium and contributes to the development of various lung pathologies [61, 62]. The ligand Wnt5a, which is secreted mostly in lung parenchymal fibroblasts, decreases the nuclear expression of β-catenin in AT2 epithelial cells and inhibits AT2-AT1 transdifferentiation, disrupting the mechanism of tissue recovery.

Alternative WNT signaling also impacts YAP/TAZ nucleocytoplasmic shuttling in cells. WNT proteins (Wnt1, Wnt5a/b, Wnt11) bind to FZD and receptor tyrosine kinase-like orphan receptor 1 (ROR1/2) coreceptors located on the plasma membrane and activate noncanonical WNT signaling via GPCR signaling, activating the LATS1/2 kinase inhibitory cascade through the WNT-FZD/ROR-Gα12/13-Rho GTPase-LATS1/2 axis, which in turn causes YAP/TAZ activation and translocation to the cell nucleus [60].

**Fig. 2:**
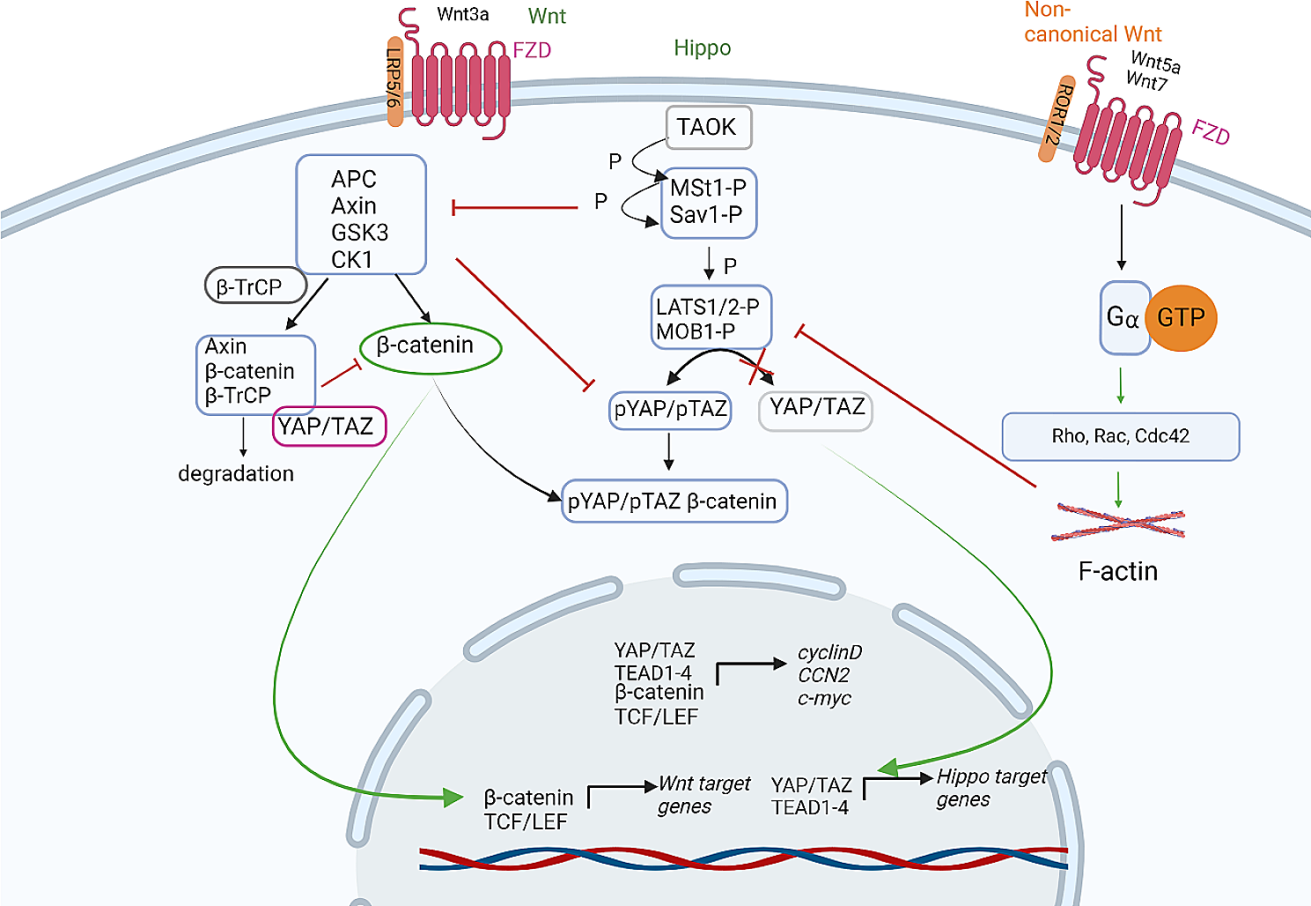
The scheme illustrates the interactions between the Hippo and WNT signaling pathways in a simplified manner. The figures were created with BioRender.com

Therefore, the core components of Hippo/YAP signaling interact with canonical and noncanonical WNT in different ways, and a detailed understanding of this interaction may contribute to the understanding of normal, pathological, and regenerative processes in the lungs and have both scientific and clinical applications. Notably, key mechanisms of Hippo-WNT interactions have been demonstrated in cell cultures in vitro, and Hippo pathway activation or inactivation also depends on cytoskeletal mechanical tension and the stiffness of the extracellular matrix. Therefore, investigating the mutual regulation of the Hippo-WNT signaling pathway in lung cells using biologically relevant models is vital and remains an unresolved task (Fig. 2).

#### TGFβ-Hippo

The transforming growth factor-β (TGFβ) superfamily of secreted polypeptide factors participates in the maintenance of normal lung homeostasis and is prevalent in the pathogenesis of pulmonary fibrosis. In published research [63], an intriguing difference between a very limited set of transmembrane serine/threonine receptors and more than 30 ligands of the TGFβ superfamily was noted. Furthermore, TGFβ ligands are highly specific and participate in different biological functions. It is possible that limited quantities of TGFβR-I and TGFβR-II receptors are compensated for by their simultaneous binding of several TGFβ ligands, and downstream processes do not induce only one or the other pathway of TGFβ pathway activation following the “all-or-none” principle; however, this theory requires further research.

Direct interactions between TGFβ and the Hippo signaling pathway have been described in the cell nucleus. Active TGFβ/Smad signaling promotes Smad nuclear translocation, where nuclear TAZ (inactive Hippo signaling) combines with the Smad2/3-Smad4 complex and controls Smad nucleocytoplasmic shuttling in human embryonic stem cells [64, 65]. In murine endothelial lung cells, active nuclear YAP, but not TAZ, interacts with Smad3 in the cell nucleus and blocks Smad3 phosphorylation and its binding to GSK3β. YAP-Smad3 complexes in cell nuclei participate in the initiation of endothelial-mesenchymal transition [66]. In human skin dermal fibroblasts, a reduction in YAP/TAZ levels results in the activation of the transcription factor AP-1, which induces Smad7 and suppresses the TGFβ/Smad3 pathway [67]. So, there is a direct dependency between YAP/TAZ activation/deactivation and induction/suppression of TGFβ/Smad signaling target genes. Additionally, TGFβ/Smad signaling can indirectly negatively regulate YAP level in lung cells via downregulation of Sox2 expression. In normal murine embryonic lungs, the TGFβ1 ligand, nuclear YAP, and Sox2 proteins are expressed in the transition zone of proximal to distal lung compartments, and the nuclear YAP expression level overlaps with that of Sox2 [7]. Moreover, there is a direct relationship between YAP and Sox2 in embryonic lungs: YAP deletion in lung epithelial progenitors leads to reduced Sox2 expression, but increased levels of nuclear YAP promote Sox2 expression. Additionally, TGFβ stimulation downregulates Sox2 and induces epithelial-to-mesenchymal transition in human lung cancer cells [68]. In addition, TGFβ inhibits Sox2-mediated epithelial-to-mesenchymal reprogramming of mouse fibroblasts. Thus, TGFβ signaling potentially participates in the downregulation of Sox2 expression in different cell types and can regulate the level of YAP, as the direct dependency of YAP-Sox2 was demonstrated in embryonic lung cells.

Noncanonical or Smad-independent TGFβ signaling can moderate YAP/TAZ intracellular localization through the modulation of cytoskeletal tension and apical‒basal polarity. The active TGFβ/non-Smad pathway induces actin reorganization via the activation of the Rho small GTPases Cdc42 and RhoA but not Rac1 [69]. Cytoskeletal alterations can lead to YAP/TAZ nucleocytoplasmic shuttling and change apical‒basal cell polarity. In polarized airway epithelial cells in mice, the polarity protein Crumbs-3 can regulate YAP/TAZ translocation into the cell nucleus [70]. In the apical domain, active TGFβ/Smad signaling causes the association of the Crumbs complex with the YAP/TAZ and Smad2/3 proteins, followed by phosphorylation in the cytoplasm, which prevents TGFβ target gene expression. In the basal-lateral domain, Scribble induces MST1/2-LATS1/2-TAZ/YAP interactions during epithelial–mesenchymal transition caused by active TGFβ/non-Smad signaling. So, noncanonical TGFβ signaling regulates Hippo signaling through Rho pathway and apical‒basal cell polarity.

**Fig. 3:**
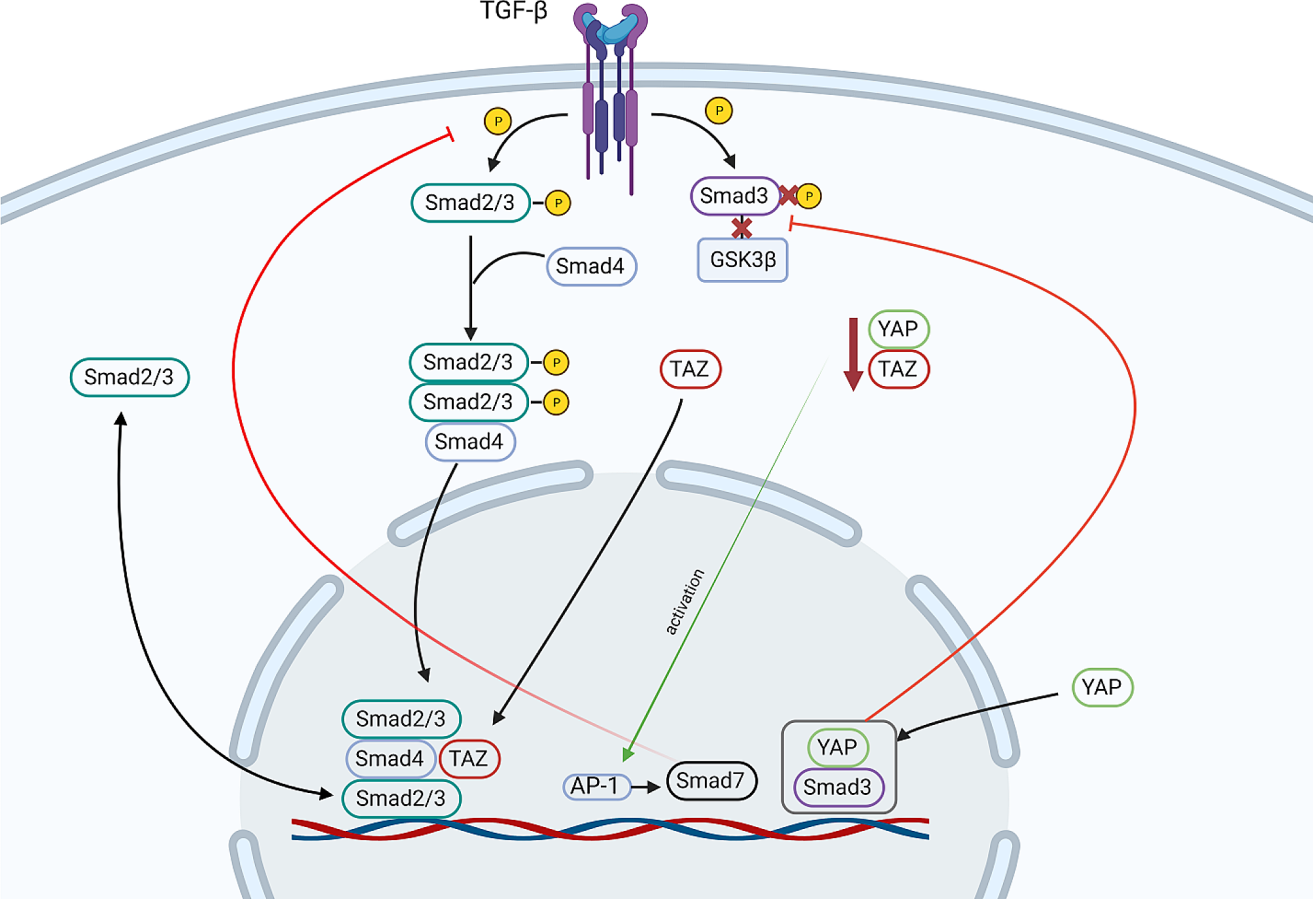
The simplified scheme illustrates the nuclear interactions between YAP/TAZ and Smads. The figures were created with BioRender.com

The described TGFβ-Hippo pathway interactions highlight the importance of both pathways in normal and pathological processes in lung cells. The lung fibroblasts of patients with idiopathic pulmonary fibrosis showed high nuclear expression of YAP/TAZ, which suggests a significant influence of YAP/TAZ on fibrosis [34, 71]. Additionally, total level of YAP was increased in AT2 and bronchiolar epithelial cells of idiopathic pulmonary fibrosis patient lungs [72]. Moreover, a central role of TGFβ signaling pathways in the development of lung fibrosis has been demonstrated [73–75]. A detailed investigation and understanding of the mutual regulation of the Hippo and TGFβ signaling pathways might allow us to propose new therapeutic approaches for the treatment of idiopathic pulmonary fibrosis (Fig. 3).

#### SHH-Hippo

During organogenesis, the Hedgehog pathway is necessary for the regulation of intercellular communication. Sonic Hedgehog (SHH) is one of the best-described ligands of the Hedgehog pathway; known orthologs of SHH include Indian HH and Desert HH. There are canonical and noncanonical, or ligand-dependent and ligand-independent, SHH pathways. The SHH signaling pathway is important for normal lung development and participates in branching morphogenesis, primary bud outgrowth, epithelial–mesenchymal interactions and the regulation of mesenchymal proliferation and differentiation. In murine lungs during several stages of early embryogenesis (E11–E16.5), SHH is clearly present in the distal tips of bronchial tubules and is weakly expressed in proximal lung compartments. Additionally, at the pseudoglandular stage, the cell surface receptors PTCH1 and GLI family proteins are highly expressed in mesenchymal cells of the distal lungs, but the SMO protein is present in epithelial and mesenchymal cells. During the late pseudoglandular and canicular stages of lung development (E16.5–E17), SHH is expressed mostly in epithelial cells of the trachea and bronchi. In the alveolarization stage, a large number of proliferating cells express the *GLI1* gene and coexpress α-SMA; it is possible that embryonic GLI1 + cells give rise to septal myofibroblasts. In the early postnatal period (P1), the bronchiolar epithelium expresses SHH; however, the levels of SHH and PTCH1 significantly decrease after birth [2]. In postnatal lung development (P1–P14), SHH plays a crucial role in alveolar myofibroblast differentiation and is necessary for mesenchymal proliferation [76]. The functional inhibition of the key components of the SHH pathway by gene knockout in perinatal development causes high mortality in animals. For example, SHH-null (-/-) and GLI2 (-/-) mice have single-lobe hypoplastic lungs with decreased epithelial and mesenchymal cells and die at birth. PTCH1 (-/-) or GLI3 (-/-) knockout in mice is lethal at the embryonic stage. Notably, knockout of different GLI family proteins has diverse consequences for animal viability. For instance, GLI1 (-/-) knockout mice exhibit normal lung development [77], but the inhibition of SHH signaling during embryogenesis blocks GLI1 cell proliferation and differentiation to myofibroblasts [76].

The SHH and Hippo signaling pathways both contribute to lung development and likely regulate each other. As demonstrated in mouse embryonic fibroblasts, the Hippo pathway-mediated LATS/TAZ/PKA/GLI3 cascade negatively regulates SHH signaling via the GLI3R protein [78]. When Hippo signaling is inactive, cytoplasmic nonphosphorylated TAZ, but not YAP, binds to PKA and phosphorylates GLI3 at conserved serine residues, resulting in GLI3 processing into GLI3R. Additionally, in cultured Hek293T cells, active YAP controls cell density to suppress SHH signaling and GLI transcription factors, and YAP knockdown in cells expressing SHH results in increased *GLI1* gene expression [79]. In cultured hepatic stellate cells, activation of the SHH pathway positively regulates YAP, which supports and controls myofibroblastic differentiation in the liver [80]. In non-small cell lung cancer (NSCLC), cell proliferation is mediated by GLI proteins [81], in particular, Gli1 knockdown reduces tumor cell proliferation and viability [82]. However, SHH ligand-independent pathway is activated in NSCLC [83], so crosstalk might occur between Hippo and non-canonical SHH pathways via GLI1 and YAP-TEAD interactions [84].

**Fig. 4:**
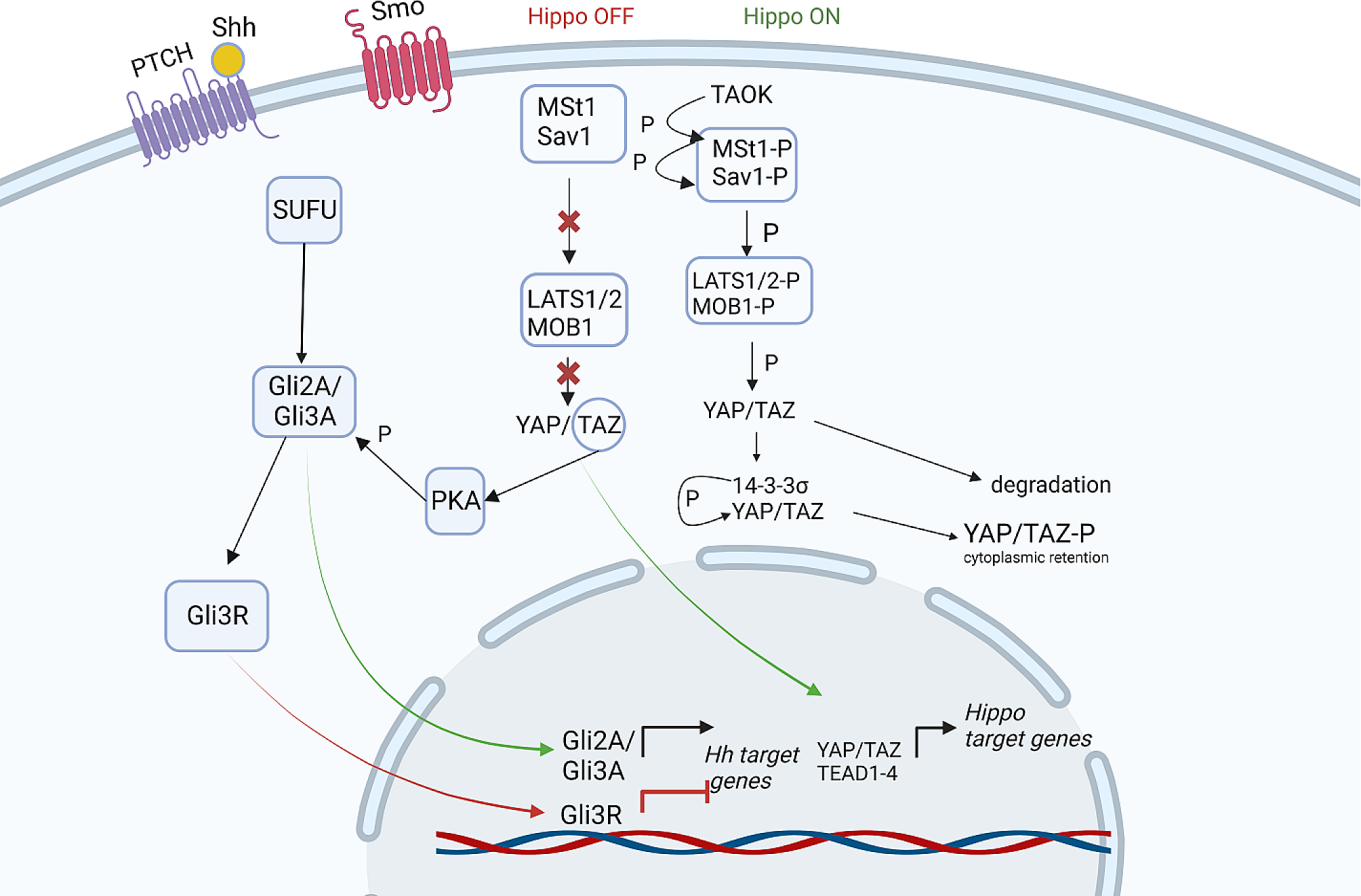
The figure presents the interactions of key components of the Hippo and SHH pathways. The figures were created with BioRender.com

Few studies have focused on the crosstalk between SHH and Hippo signaling in lung cells. However, interactions between the SHH and Hippo pathways are important for a detailed understanding of the molecular mechanisms of lung development during embryogenesis and in pathological conditions such as cancer, pulmonary fibrosis, asthma and chronic obstructive pulmonary disease. In addition, the vast majority of studies have been carried out using a monolayer of cultured cells, which is not a biologically relevant model, so the results obtained may not be translatable. During postnatal development, the SHH pathway is critical for alveolar mesenchymal differentiation, but the Hippo pathway is required for alveolar epithelial differentiation. Further research into the crosstalk between the SHH and Hippo pathways will help to improve our understanding of epithelial–mesenchymal interactions. Therefore, the mechanisms of SHH-Hippo crosstalk at different levels warrant further investigation (Fig. 4).

#### Notch-Hippo

The Notch pathway is a well-described signaling pathway involved in lung development, homeostasis and pathogenesis. Several modes of the Notch pathway have been described. First, *in the trans* modality, which refers to direct communication between receptors (Notch 1, 2, 3, and 4 receptors) expressing cells and ligands (Jagged 1 and 2 and Delta-like ligands (Dll) 1, 3, and 4) expressing cells, γ-secretase cuts the Notch intracellular domain (NICD) from the Notch receptor and promotes NICD translocation to the cell nucleus. In the nucleus, the NICD activates Notch target genes (*HES* and *HEY* family genes) through RBPJ and mastermind-like 1‐3 (Maml1‐3) transcription factors [3]. The second Notch signaling mode is *cis inhibition*, which occurs in cells with simultaneous expression of both Notch receptors and Notch ligands. The third modality is *lateral inhibition*, which results in the inactivation of Notch signaling in neighboring cells [85]. During lung development, Notch signaling participates in the regulation of cell‒cell interactions, which are pivotal for normal proximal to distal branching morphogenesis. At the pseudoglandular stage (E9.5–E16.5), Notch regulates the patterning and cell fate of epithelial progenitors. The Jagged1-Notch2 complex coordinates club and ciliated cells in developing airways [86]. Notch signaling pathway activation is necessary for club cell specification and regeneration but is not required for the self-renewal of basal cells. Additionally, Notch signaling activates the production of goblet cells and causes a decrease in the number of ciliated cells. At the canalicular stage (E16.5–E17.5), normal downregulation of Notch signaling is required for the differentiation of AT1 and AT2 cells [87], the formation of blood capillaries and the regulation of cell proliferation. From the saccular to the early postnatal alveolar phase (E17.5–P5), the Notch pathway participates in alveolarization and blood vessel development. Activation of Notch2 in AT2 cells in the neonatal period leads to paracrine activation of PDGRα signaling in alveolar myofibroblasts, leading to alveologenesis [88].

Interactions between the Notch and Hippo signaling pathways are due to nuclear YAP/TAZ interacting with Notch transcription factors to induce the expression of genes common to both the Notch and Hippo pathways. In addition to regulating Notch gene expression via the nuclear localization of YAP/TAZ, in the *in trans* modality of the Notch pathway, YAP/TAZ can inhibit Notch signaling via the *cis-mediated inhibition* of Notch ligands and receptors. Therefore, Hippo–Notch crosstalk is context dependent, and inactive Hippo can result in inactive Notch through *cis inhibition*. At the same time, active Notch can be directly triggered by nuclear YAP/TAZ via the *in trans* modality of the Notch pathway. As shown in the human embryonic pancreas, the YAP-TEAD complex in the cell nucleus can bind to *HES1*, which leads to the repression of endocrine cell differentiation [89]. In neural crest cells, nuclear YAP binds to the NICD-RBPJ transcription factor to control smooth muscle cell differentiation [90]. In early embryonic development, the blastocyst YAP-TEAD and NICD-RBPJ complexes jointly regulate the expression of the trophectoderm master gene *CDX2*, but in these cells, differentiation into the trophectoderm occurs independently of the Hippo/YAP and Notch signaling pathways [91]. In epidermal basal cells, nuclear YAP activates the Dll1 ligand, which results in active Notch signaling in neighboring cells and induces cell differentiation [85].

In lung cells, interactions between the Hippo and Notch signaling pathways are poorly described. Similar to the Hippo signaling pathway, the Notch pathway plays a crucial role in airway cell differentiation during embryogenesis. In particular, Notch overexpression is required for club and goblet cell differentiation. It is likely that at this stage of airway development, there is an active interaction of the Hippo-Notch signaling pathway, resulting in the normal development of proximal lung compartments. Moreover, active YAP is necessary for the normal development of distal lung compartments [18], but Notch overexpression downregulates the differentiation of distal progenitors into alveolocytes [87, 92]. In addition, the role of Notch signaling in both the development of mesenchymal progenitor cells and regeneration mechanisms is still unknown. Therefore, the study of Hippo-Notch pathway interactions in lung cells during embryogenesis and in both normal and pathological adult lungs is a very promising research direction.

#### mTOR-Hippo

Mammalian target of rapamycin (mTOR) is a serine/threonine kinase that is part of an intracellular metabolic pathway that controls proliferation, epithelial cell behavior, nutrient and energy availability and morphogenesis. Dysregulation of mTOR signaling in the lungs results in different pathologies, such as idiopathic pulmonary fibrosis, COPD, pulmonary hypertension, lymphangioleiomyomatosis and other lung cancers [93–96].

Two multiprotein complexes with opposing sensitivities to rapamycin have been described: mTOR1 (sensitive), which combines with the regulatory-associated protein Raptor, and mTOR2 (resistant), which then binds to the rapamycin protector Rictor. mTOR1 and mTOR2 complexes are both induced by intracellular and extracellular factors such as growth factors, the extracellular matrix, and hormones. Additionally, mTOR1 can be stimulated through energy and cellular stress, adenosine monophosphate (AMP), adenosine triphosphate (ATP), and translation inhibition. Additionally, amino acids are able to positively regulate mTOR1 through Rag GTPase. Energy stress leads to tuberous sclerosis complex (TSC1/TSC2)-dependent mTOR1 inhibition through AMP-activated protein kinase (AMPK), which phosphorylates Raptor and binds phospho-Raptor with the 14-3-3 sigma protein [97]. For mTOR2 pathway activation, growth factors and insulin stimulate the PI3K pathway and activate AKT through Rho GTPases [98]. The GSK3 protein can phosphorylate Rictor to inhibit mTOR2 activity and phosphorylate TSC2 to increase mTOR1 activity [99]. Therefore, the molecular mechanisms regulating mTOR1 and mTOR2 are quite different and, in the case of mTOR2 signaling, are still poorly described.

In lung morphogenesis, the mTOR signaling pathway participates in the regulation of alveologenesis by controlling mitochondrial function [100]. For instance, in the postnatal period (P10), Raptor inactivation in mice leads to the loss of mitochondria and alveolar lung defects. Additionally, in fetal lungs, airway and vascular branching morphogenesis is regulated by the interaction between mTOR1, hypoxia-inducible factor (HIF) and VEGF-A pathways [101].

In mouse lungs, continuous activation of YAP/TAZ induces the mTOR-ATF4 signaling pathway and leads to changes in the cell fate and identity of secretory airway cells and their conversion into squamous AT1-like cells [102]. In human bronchial epithelial cells (HBEC3s) an active interaction was found between YAP and mTOR/PI3K/AKT pathway, resulting to increased cell proliferation, migration and polarity, that potentially contribute to development of the idiopathic pulmonary fibrosis [72]. Raptor deletion in YAP/TAZ-activated lung cells offsets the loss of secretory cell identity and mTOR1 and ATF4 activation. The interaction between the Hippo/YAP and mTOR signaling pathways can be achieved through the upregulation of microRNA-29 and downregulation of phosphatase and tensin homolog (PTEN), which are negative regulators of the PI3K-AKT and mTOR cascades, respectively [103]. As demonstrated in vitro in human lung adenocarcinoma cells, YAP regulates proliferation via the PTEN/AKT/mTOR signaling pathway; specifically, YAP inhibits PTEN and activates the AKT/mTOR pathway [104]. Furthermore, in HEK293 and MCF7 cells, LATS1/2 kinase was shown to phosphorylate Raptor at Ser606 and negatively regulate mTOR1 activation [105]. In malignant glioma cells, MST1/2 kinase binds to AKT and deactivates the mTOR pathway [106]. Additionally, Hippo pathway-related long noncoding RNAs (lncRNAs) are involved in the coactivation of the mTOR1 and Hippo signaling pathways in breast cancer, cholangiocarcinoma and colon cancer [107]. 

**Fig. 5:**
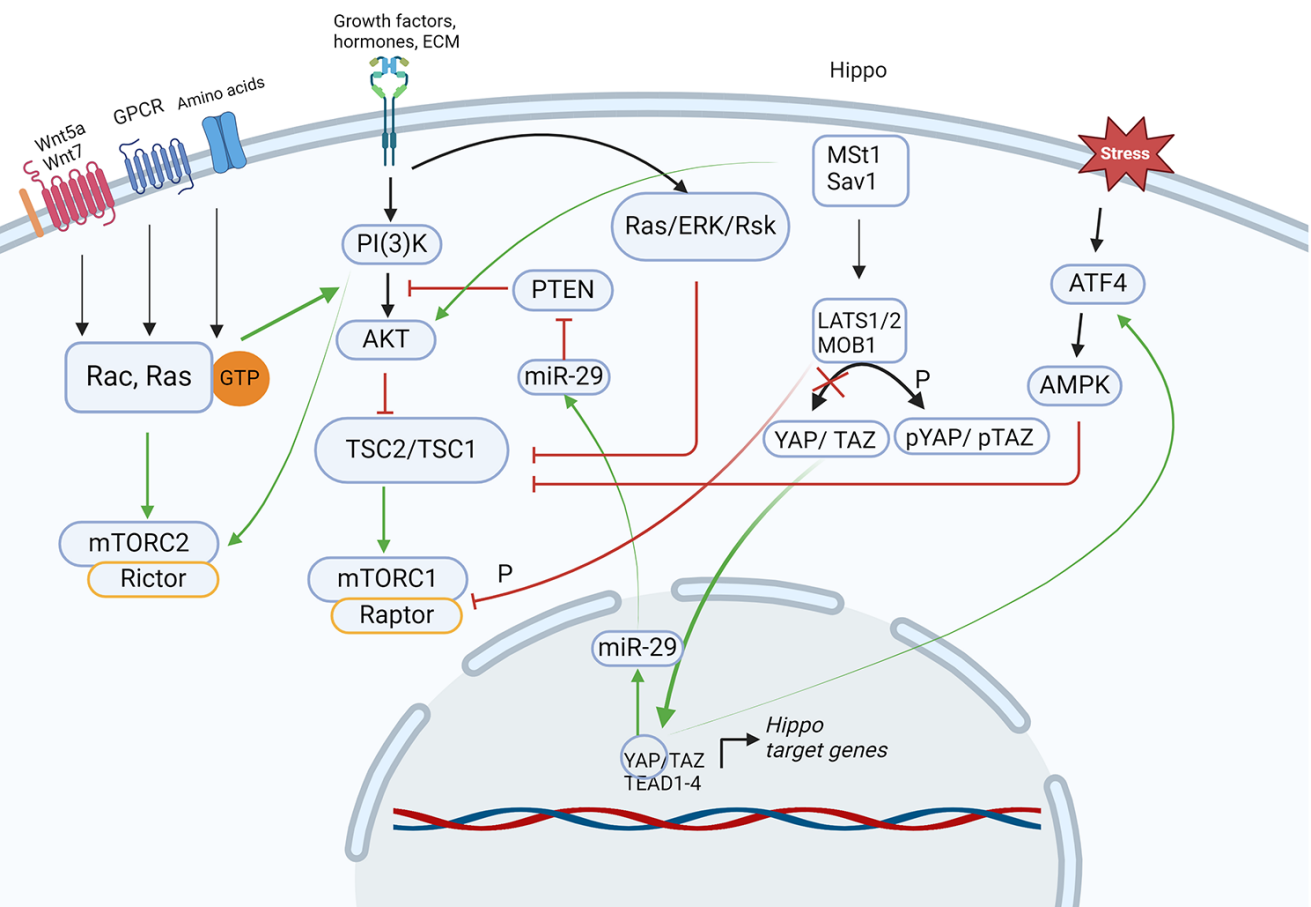
The scheme illustrates the interactions between the Hippo and mTOR signaling pathways in a simplified manner. The figures were created with BioRender.com

Cellular metabolism, energy deficit and Hippo signaling are interdependent and can be mutually regulated (Fig. 5). However, the molecular mechanisms underlying the interactions between mTOR and the Hippo pathway have been studied in specific cell lines in vitro [104–107]. In addition, the mechanisms regulating the mTOR2 pathway in the context of Hippo signaling crosstalk remain practically unexplored. Activation of both the mTOR2 and Hippo signaling pathways is possible through Rho GTPases. Therefore, investigating the molecular interactions between the mTOR and Hippo signaling pathways in biologically relevant models is a current and unresolved task.

## Conclusion

The study of regenerative and pathological processes at the level of signaling cascades in airway cells is vitally important, as respiratory diseases are the second most common cause of death in the world after vascular diseases, according to the WHO [108]. The Hippo signaling pathway is involved in the regulation of cell proliferation, differentiation and self-renewal at all stages of airway development and in the adult lung (Table 2). Despite numerous studies dedicated to studying Hippo signaling crosstalk with other pathways, relatively few of those aimed to explore this crosstalk in lung cells. The interaction and mutual regulation of Hippo signaling by other signaling pathways in lung cells is necessary for normal development, organ function and maturation of regeneration processes. In the lung, the WNT, Notch, SHH, Rho, TGFβ, and mTOR signaling pathways are crucial for the lung and have overlapping functions in embryogenesis and in adulthood (Table 2). Intersignaling crosstalk in cells is governed by complex and finely tuned interaction mechanisms between different signaling components in the cytoplasm, nucleus, and extracellular space. Elements of the Hippo cascade interact with distinct members of the WNT, Notch, SHH, Rho, TGFβ, and mTOR pathways, which determine the intracellular localization of YAP/TAZ transcriptional cofactors of the Hippo pathway. Thus, dysregulation of one signaling pathway leads to dysregulation of other signaling cascades, creating a “snowball” effect.

**Table 2:** Common functions of the described signaling pathways at different developmental stages in normal murine lungs

	Hippo	Wnt	TGFb/BMP	Shh	Notch	mTOR
Embryonic (E9.5-E12.5) and pseudoglandular stage (E9.5–E16.5)	Branching morphogenesis; development of alveoli, bronchiolar departments	Generation mesoderm cells; branching morphogenesis; endothelial differentiation; tracheal development	Branching morphogenesis; development of alveolar, bronchiolar departments	Expressed in epithelium and mesenchyme	Regulation of the patterning and cell fate of epithelial progenitors; club cell specification	Branching morphogenesis;
Canalicular stage (E16.5–E17.5)	Branching morphogenesis; control basal cell differentiation	Alveolar cell differentiation	Epithelial growth and differentiation; induce epithelial–mesenchymal transition	Trachea and bronchi development	Alveolar cell differentiation	Branching morphogenesis;
Saccular stage (E17.5-P5)	Alveolarization; regulation of proliferation and differentiation of airway epithelial cells	Alveolar cell differentiation	Induction epithelial–mesenchymal transition	Trachea and bronchi development	Alveolarization;	Saccule formation; alveolar cell differentiation
Alveolar stage (P5 -P30)	Alveolarization; regulation of proliferation and differentiation of airway epithelial cells; control of AT2-AT1 differentiation	Regulation of alveologenesis, control of AT2-AT1 differentiation	Regulation of alveologenesis, control of AT2-AT1 differentiation	Alveolar myofibroblasts differentiation; mesenchymal proliferation	Alveolarization	Regulation of alveologenesis,
Adult lungs	Maintaining homeostasis; alveolar self-renew after injury	Maintaining homeostasis; regeneration after injury	Maintaining homeostasis	Maintaining homeostasis	Maintaining homeostasis	Maintaining homeostasis

One of the major problems in studying signaling interactions is that almost all studies of the mechanisms by which components of specific signaling pathways interact with each other are carried out in vitro on cells grown in a monolayer. It is well known that culture conditions directly influence processes in cells, particularly in the context of YAP/TAZ, which are mechanotransducers. In vitro processes in cells can differ significantly from those in vivo, especially for delicate regulatory mechanisms such as intersignaling crosstalk. Therefore, the problem of how Hippo interacts with other signaling pathways in more biologically relevant models in vivo remains unresolved. In addition, most related research has focused on processes in lung epithelial cells, with only a small number of studies focusing on mesenchymal cells, which are components of the epithelial microenvironment. Currently, an increasing number of studies on the specification of mesenchymal cell lineages in the lung are highlighting the important role of different mesenchyme types in epithelial differentiation. The role of intracellular YAP/TAZ localization in different mesenchyme types and how components of other signaling pathways influence YAP/TAZ nucleocytoplasmic shuttling are currently poorly described.

Importantly, omics technologies promise to significantly expand and deepen our knowledge of intra- and intercellular interactions. Single-cell RNA sequencing combined with spatial transcriptomics enables the understanding of regulatory networks, signaling pathways, and cell‒cell communications [109]. In addition, the development and improvement of methods and systems for single-cell data analysis have enabled the construction of classification pathway databases for the study of signaling interactions [59, 110–112]. In view of the complexity and numerous multifactorial processes involved, the determination of YAP/TAZ nucleocytoplasmic shuttling in living cells is likely to require deep data analysis, including biophysics, machine learning, and artificial intelligence, which should be considered in future research.

## Data Availability

No datasets were generated or analysed during the current study.
